# Fibroblastic and myofibroblastic tumors of children: new genetic entities and new ancillary testing

**DOI:** 10.12688/f1000research.16236.1

**Published:** 2018-12-20

**Authors:** David M Parham

**Affiliations:** 1 Pathology and Laboratory Medicine, Children's Hospital Los Angeles, Los Angeles, CA, USA; 2 Department of Pathology, USC Keck School of Medicine, Los Angeles, CA, USA

**Keywords:** fibrous hamartoma of infancy, infantile myofibromatosis, lipofibromatosis, infantile fibrosarcoma, primitive myxoid mesenchymal tumor of infancy, spindle cell rhabdomyosarcoma

## Abstract

Fibroblastic and myofibroblastic tumors comprise a morphologically diverse and biologically variable group of neoplasms that affect a wide age range. Specific entities tend to occur most frequently in infants and young children. Recent years have witnessed a proliferation of information concerning the unique biology of these tumors. In this report, I will review recent findings that serve to further characterize this group of neoplasms. Included will be newer information on fibrous hamartoma of infancy, infantile myofibromatosis, lipofibromatosis, and infantile fibrosarcoma and tumors resembling it, including primitive myxoid mesenchymal tumor of infancy and new genetic entities. I will also discuss the differential diagnosis, which includes spindle cell rhabdomyosarcoma, dermatofibrosarcoma protuberans, and calcifying aponeurotic fibroma.

## Introduction

Pediatric soft tissue tumor pathology comprises a wide variety of morphologic and genetic entities. Recent advances in genetic techniques and investigations during the past 10 years have resulted in changes and updates in the diagnosis of infantile fibroblastic and myofibroblastic tumors. These updates include genetic correlates of infantile myofibroma (IM) and fibrous hamartoma and affect an array of spindle cell tumors formerly included within infantile fibrosarcoma (IF) and fibromatosis. These new data have led to the development of new effective therapies using targeted agents. This new therapy behooves the pathologist to become facile in recognizing these entities, ordering appropriate ancillary testing, and recommending personalized treatment. In this article, I will describe updates and new findings involving fibrous hamartoma of infancy (FHI), infantile myofibromatosis, lipofibromatosis (LPF), IF, primitive myoid mesenchymal tumor of infancy, and spindle cell rhabdomyosarcoma (SCRMS).

## Fibrous hamartoma of infancy

**Figure 1.**
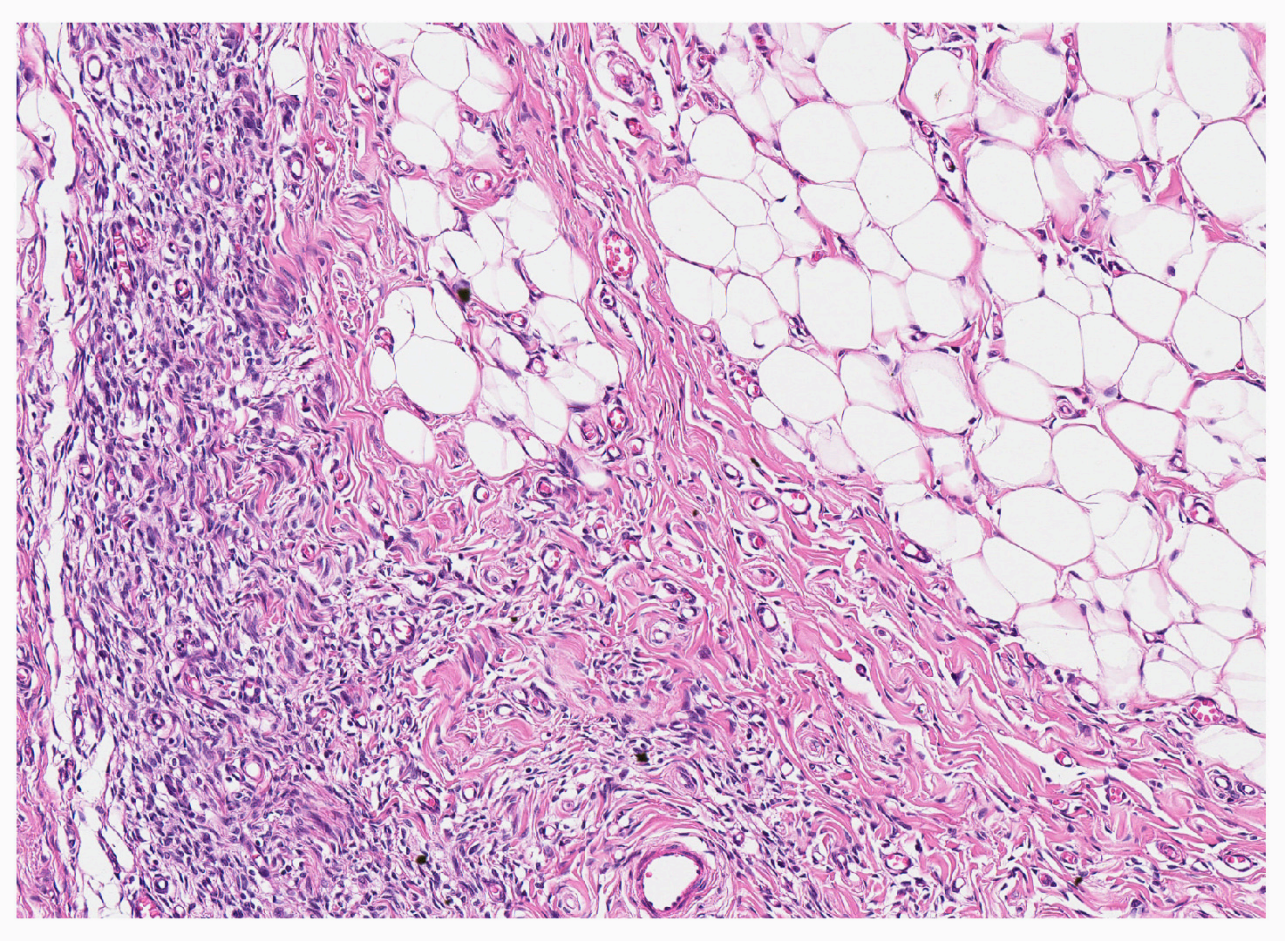
Fibrous hamartoma of infancy. A mixture of immature mesenchyme, mature fibroblasts, and adipose tissue is arranged in a haphazard organoid fashion. Hematoxylin and eosin stain, with scale bar. This figure uses an original image taken by DMP for publication.

FHI is generally easily recognizable by its triphasic content of primitive mesenchyme, myofibroblasts, and mature fat, arranged in an organoid pattern ( Figure 1). They most commonly arise within the axilla, groin, upper extremities, and back, forming subcutaneous plaques that are easily excised. Recurrences are rare, even with simple excision.

**Figure 2.**
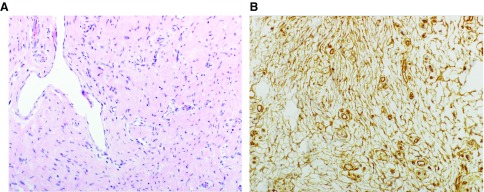
Fibrous hamartoma of infancy. This lesion contains areas with a pseudoangiomatous giant cell fibroblastoma-like pattern, as shown by the dilated vessels and dense collagen ( **A**). CD34 expression is found in this area ( **B**). ( **A**) Hematoxylin and eosin, 40× objective. ( **B**) CD34 immunostain, 20× objective. This figure uses an original image taken by DMP for publication.

FHI has been the subject of two large clinicopathological reviews. A review of 60 patients with FHI by Saab *et al*. ^1^ reported an unusual pseudoangiomatous pattern in about half of the cases ( Figure 2A, B). Immunohistochemistry (IHC) revealed reactivity for smooth muscle actin (SMA) and CD34 in the mature fibrous tissue and variable CD34 expression in the primitive mesenchyme. Of 12 cases with follow-up, two recurred. That article highlighted an expanded age range (up to eight years) and anatomic location (breast, distal extremities, and head and neck). Of note, Ki67 staining revealed that cellular proliferative index was high (up to 100%, mean 15%) in the immature mesenchyme in some cases.

Al-Ibraheemi *et al*. ^2^ published a larger series of 145 cases, including cases from the Mayo Clinic, Emory, Boston Children’s Hospital, and Children’s Hospital Los Angeles. Of particular interest in this series were two cases with sarcomatous features. These two lesions contained highly cellular spindle and round cell foci with mitotic activity and abruptly appeared as focal nodules within an otherwise typical FHI. In one case, an osteoid-like matrix was observed. Genetic findings in these two lesions included (1) hyperdiploid/near triploid karyotype with chromosome gains and loss of heterozygosity of chromosomes 1p and 11p and (2) loss of 10p, 14, and a large portion of 22q (22q11.23q13.33). Otherwise, the features described by Al-Ibraheemi *et al*. resembled those seen by Saab *et al*. ^1^ with a similar expansion of anatomic sites and age range (up to 14 years). Of note was the presence of giant cell fibroblastoma (GCF)-like areas ( Figure 2A, B), which were CD34 ^+^ ( Figure 2C) and seem to correspond to the “pseudoangiomatous” pattern of Saab *et al*. ^1^. In cases with follow-up, there were two recurrences and no metastases.

**Figure 3.**
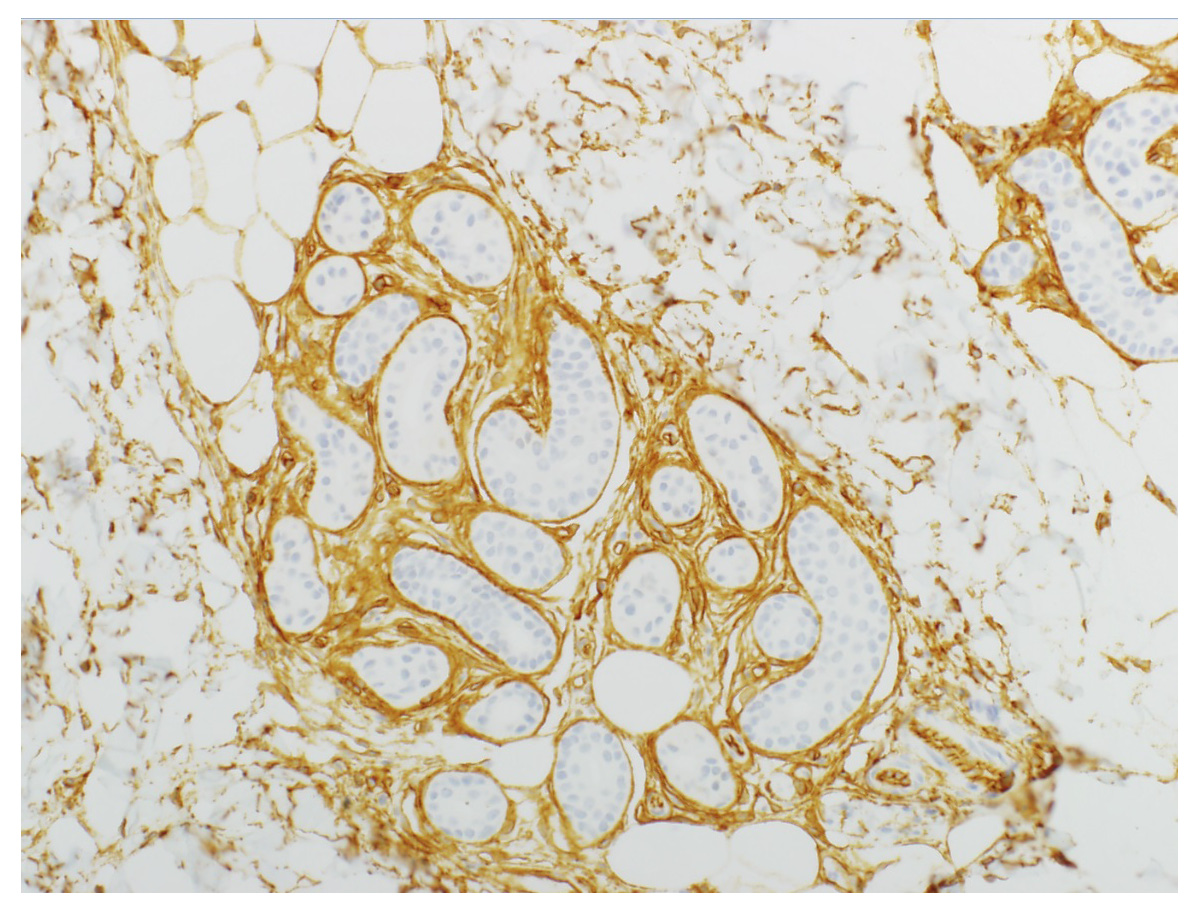
CD34 expression is found in the mesenchyme surrounding skin adnexae. CD34 immunostain, 20× objective. This figure uses an original image taken by DMP for publication.

A landmark genetic finding in FHI was published in 2016 by Park *et al*. ^3^. That article described the discovery of epidermal growth factor receptor ( *EGFR*) exon 20 insertion/duplication mutations in 12 cases of FHI, casting a cloud over their status as true “hamartomas” and suggesting that the distinction from neoplasia may be arbitrary. This discovery was made by using next-generation sequencing (NGS) that included an array of commonly occurring driver mutations. IHC for EGFR in this cohort of cases revealed minimal to moderate staining, mostly within primitive mesenchyme. EGFR is important to hair differentiation ^4^, suggesting a possible link of FHI to the CD34 ^+^ mesenchyme that surrounds the skin adnexae ( Figure 3) and explaining their peculiar anatomic distribution, but more research is needed on this point.

## Infantile myofibroma

**Figure 4.**
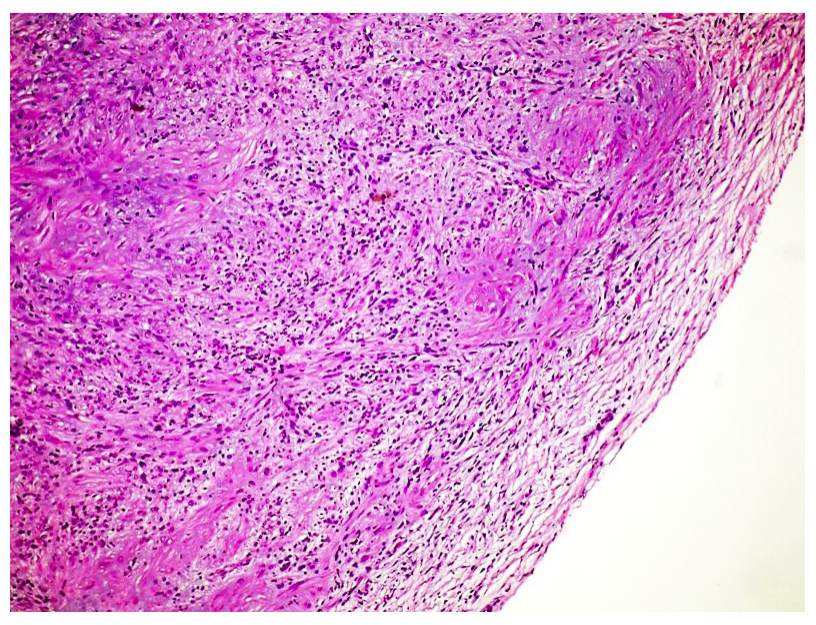
Infantile myofibroma. Alternating areas of mature myofibroblastic cells and immature mesenchyme with a hemangiopericytoma pattern comprise this lesion. Hematoxylin and eosin, 20× objective. T This figure uses an original image taken by DMP for publication.

IM is classically a biphasic neoplasm with a hemangiopericytoma (HPC)-like core and a myofibroblastic shell ( Figure 4). This entity was included as simply “myofibroma” in the 2002 World Health Organization (WHO) lexicon and comprised part of the morphological spectrum of “myopericytoma, including myofibroma” in the 2013 version, albeit as a distinct non-synonymous entity with overlapping features. However, “infantile myofibroma” well describes its usual clinical presentation in pediatrics and continues to see common usage. The term has also subsumed lesions that were once called “infantile HPC”, reflecting the variability in the relative content of HPC and myofibroblasts. In many examples, the HPC-like foci form the chief component; in others, the myofibroblast content predominates.

IM may occur as solitary or multifocal lesions, for which the term “myofibromatosis” is used. They are usually small, well-circumscribed tumors, but some cases may be large and fibrosarcoma-like in presentation, engendering the term “composite fibromatosis” ^5^. In a series reviewed by Alaggio *et al*. ^5^, seven composite tumors were studied. These were highly cellular tumors with diffuse growth of primitive spindle cells resembling IF, combined with foci more typical of IM. Four cases were classified as IF, but the *ETV6-NTRK3* fusion was absent. These lesions indicated a morphologic continuum between IF and IM and highlighted the importance of genetic testing for diagnosis. Newer findings described below may include these lesions.

Another feature of IM is its tendency to involve multiple soft tissue and skeletal sites. In the generalized form, multiple viscera can be involved. In one recent series of 25 patients ^6^, the solitary form of IM accounted for 14 cases and the multicentric form for 11 cases. None of the solitary lesions recurred following excision. One patient had a constitutional 6q deletion associated with dysmorphic features and mental retardation. Five of the 11 multicentric patients belonged to the same family, indicative of autosomal dominant inheritances. Bony involvement occurred in 6 out of 11. The nodules spontaneously regressed in 7 patients who received no treatment. Three patients, none with a family history, had the congenital generalized form: gastrointestinal, hepatic, cardiac, pancreatic, osseous, and pulmonary lesions were found in one patient, who died presumably from cardiac disease. The other two patients showed complete spontaneous remission, but one had a metastatic rhabdomyosarcoma at two years of age.

In 2017, Agaimy *et al*. ^7^ described recurrent *PDGFRB* mutations in sporadic infantile and adult myofibromas but not in angioleiomyomas and myopericytomas, based on mutations found in congenital inherited IM. In a follow-up study, Arts *et al*. ^8^ sequenced *PDGFRB* in a series of 16 IM samples of sporadic IM. Mutations in the coding sequence of *PDGFRB* were found in six out of eight cases of multicentric IM and one out of eight unifocal IM. These mutations variably affected exons 18, 14, 12, and 11 and constitutively activated the receptor, as demonstrated by luciferase reporter assay. The results point to the use of tyrosine kinase inhibitors for treatment of severe disease.

Other abnormalities found in a novel subset of cellular myofibromas include *SRF-RELA* fusions, as described in 2017 by Antonescu *et al*. ^9^ After discovery of *SRF-C3ORF762* and *SRF-RELA* fusions in two index cases of pediatric myofibromas, the authors found eight cellular myofibromas with *SRF-RELA* fusions among a cohort of 42 similar cases. These lesions displayed typical IM features, including multinodular growth with a biphasic myoid and cellular HPC phenotype, with co-expression of for SMA and desmin but negativity for myogenin. Only one patient experienced recurrence and he is now disease-free 44 months after the histologic diagnosis.

## Infantile fibromatosis/lipofibromatosis

LPF was first described in 2000 by Fetsch *et al*. ^10^, although similar lesions were included as “infantile fibromatosis” in older editions of Enzinger and Weiss’s standard textbook ( *Enzinger and Weiss’s Soft Tissue Tumors*). The latter term persists in their most recent (6th) edition but with LPF added in parentheses. Needless to say, a lot of nosologic uncertainty seems to persist with these lesions, and “infantile fibromatosis” and “lipofibromatosis” appear to overlap. LPF appears to be unique to children, whereas the other tumors comprise pediatric examples of adult fibromatoses.

**Figure 5.**
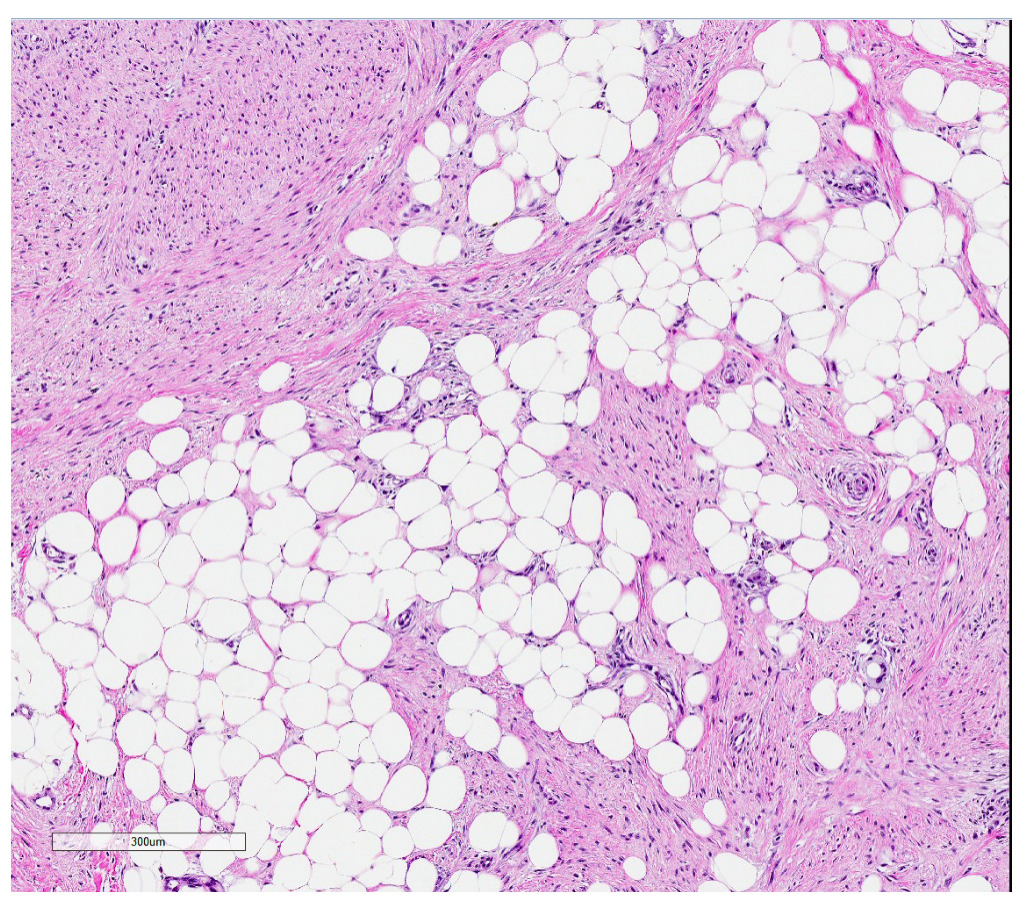
Lipofibromatosis. This lesion contains a mixture of mature fat and well-differentiated myofibroblasts. Hematoxylin and eosin, scale bar attached. This figure uses an original image taken by DMP for publication.

Like adult fibromatosis, LPF is an infiltrative and poorly circumscribed tumor. There is considerable variation in its morphological appearance, reflecting variable maturation of the constituent fibroblasts. LPF predominantly arises in infants and comprises rounded to oval to spindled cells that are intimately associated with myofibers and adipocytes ( Figure 5). The interspersed adipocytes may be extensive and many feel that the adipocytes are entrapped rather than neoplastic. Univacuolated adipocytes may be seen at the fatty–fibroblastic interface, suggesting a transitional form. Some lesions are highly cellular and mitotically active, suggesting a diagnosis of IF. Focal reactivity may be noted for CD99, SMA, CD34, and less frequently S100 and epithelial membrane antigen ^10^.

In 2016, Agaram *et al*. ^11^ reported a series of LPF-like cases with variable atypia and S100 and CD34 reactivity, which they termed “lipofibromatosis-like neural tumors” (LPF-NT). Using the FusionSeq technique, the authors were able to demonstrate unique *TPR1-NTRK1* and *TPM3-NTRK1* gene fusions in these lesions. In an archival series of 14 cases, 10 showed NTRK1 rearrangement by fluorescence *in situ* hybridization (FISH). The *LMNA-NTRK1* fusion resulted from an interstitial deletion (0.7 Mb apart) on 1q22, and in four cases there was inversion. NTRK1, being present in 9 of the 10 cases, was shown to be a sensitive marker for LPF-NT. In contrast, no *NTRK1* rearrangements were found in 25 classic LPF, suggesting this to be a unique tumor.

Another new entity resembling fibromatosis or LPF has been newly described by Kao *et al*. ^12^ and Michal *et al*. ^13^ as “ *EWSRI-SMAD3*-rearranged fibroblastic tumor” (ESFT). These tumors have occurred in patients who are 1 to 68 years old; two occurred in young children (one and five years of age). ESFT shows a lobulated or plexiform appearance, an infiltrative growth pattern, and cells with a moderate amount of eosinophilic cytoplasm. Hyalinized areas are a distinctive feature, intermingling with the more cellular component and forming a zonation pattern. Immunohistochemical testing of ESFT reveals a distinctive ERG positivity, and FISH of tested cases showed *EWSR1* rearrangement. Fusion studies with NGS revealed an *EWSR1-SMAD3* fusion. ESFT are superficial lesions that may involve the dermis, and incompletely excised lesions have a capacity for local recurrence.

## NTRK tumors/infantile fibrosarcoma

Among soft tissue lesions, *NTRK1* fusions are not limited to LPF-NT. In 2016, Haller *et al*. ^14^ reported the discovery of an *LMNA-NTRK1* fusion in four patients with an HPC-like sarcoma, including two infants and two adults. The pediatric cases resembled IM. All cases showed dysplastic appearing vessels with myxohyaline myointimal proliferations of myofibroblasts. IHC revealed CD34 and SMA expression. NGS testing of a similar adult tumor led to the discovery of another *LMNA-NTRK1* fusion-associated lesion ^15^. Importantly, this patient’s tumor and metastases showed a dramatic response to LOXO-101, a specific TRK inhibitor.

**Figure 6.**
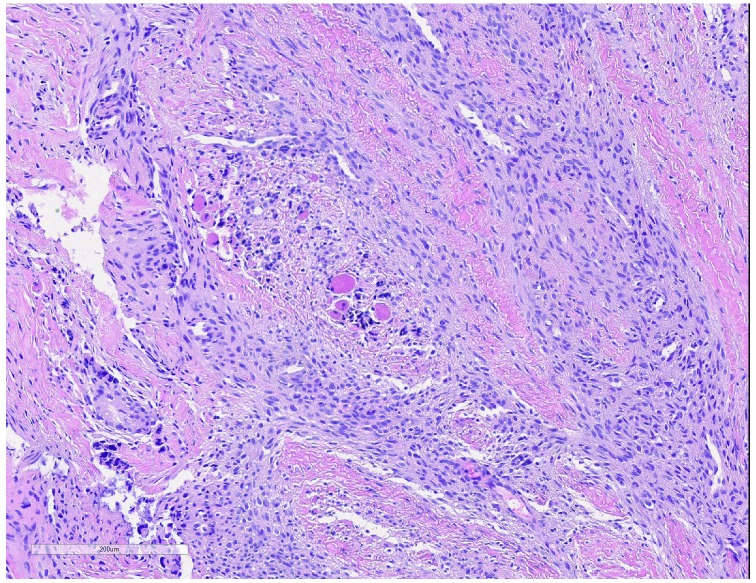
NTRK1 fusion sarcoma. This is a newly described entity related to infantile fibrosarcoma. The lesion infiltrates entrapped skeletal muscle and shows a herringbone pattern of crisscrossing neoplastic fibroblasts. The cellularity is high, and there is a moderate amount of intercellular collagen. This figure uses an original image taken by DMP for publication.

Davis *et al*. ^16^ similarly found NTRK fusions in a study of six IF-like cases lacking *ETV6-NTRK3* fusions and studied by NGS. Four of these tumors contained TMP1-NTRK1 fusions, one a *LMNA-NTRK1* fusion and one a variant *EML4-NTRK3* fusion. These tumors showed infiltrative IF- or LPF-like morphology ( Figure 6), and CD34 and CD30 were commonly expressed; SMA and S100 were variable. None showed strong S100 expression, unlike the LPF-NT described by Agaram *et al*., but all stained for NTRK1. In a follow-up study, Rudzinski *et al*. ^17^ evaluated Pan-Trk IHC in a series of 79 pediatric soft tissue tumors. Thirty tumors demonstrated *EVT6* or *NTRK* rearrangement, and negative controls included 50 cases of non- *NRTK*-rearranged pediatric soft tissue lesions. Among this cohort, there was 97% sensitivity and 98% specificity for Pan-Trk, compared with 100% sensitivity but 63% specificity for NTRKA (the protein product of NTRK1).

Laetsch *et al*. ^18^ published results of a phase 1 trial of larotrectinib, a selected inhibitor of TRK kinases; 14 out of 15 patients with tumors containing documented TRK fusions showed encouraging antitumor activity, and the compound was well tolerated.

## Primitive myxoid mesenchymal tumor of infancy

**Figure 7.**
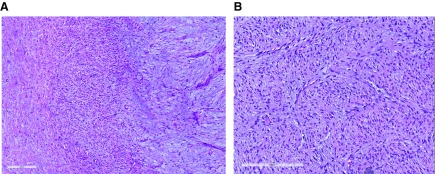
Primitive myxoid mesenchymal tumor of infancy. ( **A**) This lesion contains fields of alternating cellularity and has highly cellular areas resembling fibrosarcoma and hypocellular fields containing abundant myxoid stroma. ( **B**) This image highlights a prominent arcuate vascularity. Hematoxylin and eosin, scale bars attached. This figure uses an original image taken by DMP for publication.

In 2006, Alaggio *et al*. ^19^ reported six cases of a previously unreported infantile tumor (three congenital) with infiltrative soft tissue masses of the trunk, extremities, and head and neck. These were IFS-like but were negative for *ETV6-NTRK3* fusion. They contained a diffuse proliferation of spindle, polygonal, and round cells in a myxoid stroma. They exhibited variable degrees of cellularity, vague nodularity and collagenization, and a delicate vascular network ( Figure 7). Of five patients with follow-up, three had persistent or recurrent disease. The authors proposed classifying these lesions as a new distinct entity, “primitive myxoid mesenchymal tumor of infancy” (PMMTI).

**Figure 8.**
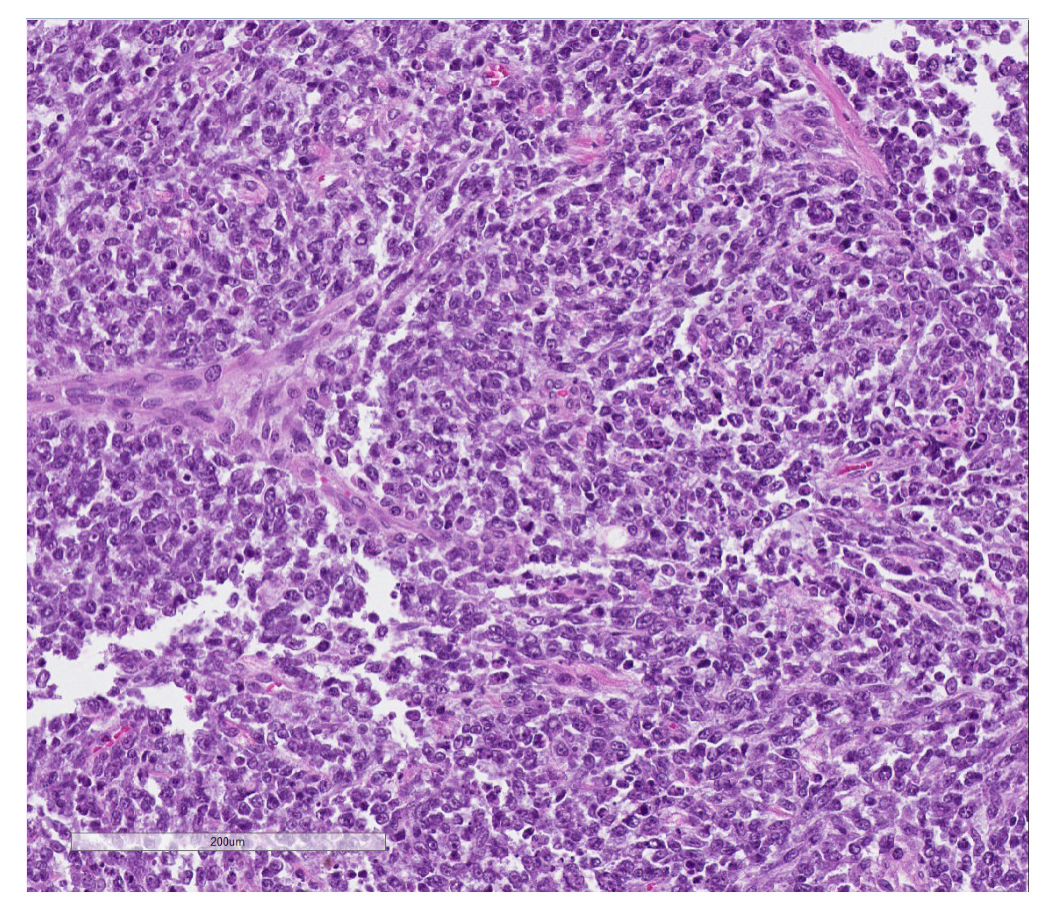
Undifferentiated round cell sarcoma of infancy. This highly cellular primitive sarcoma shows a prominent arcuate vascularity among sheets of small round undifferentiated tumor cells. BCOR immunostaining showed nuclear positivity. This figure uses an original image taken by DMP for publication.

Only sporadic reports of PMMTI appeared for the next 10 years and it seemed that this was a very rare tumor ^20^. Then in 2016, Kao *et al*. ^21^ published a recurrent genetic finding linking PMMTI with clear cell sarcoma of the kidney (CCSK) and undifferentiated round cell sarcomas (URCSs) of infants ( Figure 8). Recurring internal tandem duplication (ITD) of *BCOR* exon 16 was found in nine out of 22 URCS and six out of seven PMMTI as well as three out of four CCSK tumors. All of these lesions shared a prominent capillary network as seen in the index cases of PMMTI, with variable degrees of myxoid change, and all cases contained uniform small round cells. PMMTI was thus touted as a possible soft tissue version of CCSK, the lesion originally described as “sarcomatous Wilms tumor”.

A follow-up IHC study tested BCOR expression in soft tissue and renal sarcomas ^21^. Of 18 cases with *BCOR* ITD, 17 were positive for BCOR and 10 for SATB2. BCOR and SATB2 expression was also reported in most cases with *BCOR* or *YWHAE-NUTM2B* fusions and was seen in about one third to one half of synovial sarcomas. Occasional rhabdomyosarcomas, myxofibrosarcomas, and non-BCOR Ewing or Ewing-like sarcomas were also positive, limiting the utility of these markers without genetic confirmation. However, identification of *BCOR* ITD distinguishes PMMTI from IFS, as shown by Santiago *et al*. ^22^ in a comparison study with 11 ETV6-rearranged sarcomas, none of which expressed BCOR by IHC. BCL6 was also used as a confirmatory marker since it is a molecular target of BCOR. Of note, however, is that diffuse TRK expression has been reported in PMMTI ^23^, limiting that marker’s use for distinguishing the two lesions.

## Other new findings in translocation-negative infantile fibrosarcoma

Other new entities that have recently emerged from the study of translocation-negative IFS include those with recurrent *BRAF* fusions or *TFG-MET* fusions. In spite of their different molecular profile, these lesions appear to lie within the clinical and pathological spectrum of IFS.

In 2018, Kao *et al*. ^24^ first encountered a retroperitoneal tumor morphologically containing long intersecting fascicles, scattered lymphoplasmacytic infiltrates, and dilated vascular channels. IHC disclosed only patchy staining for SMA and caldesmon and negativity for a wide array of other markers. Based on these results and a negative FISH battery, a diagnosis of “unclassified low grade spindle cell sarcoma” was initially rendered. Targeted RNA sequencing (RNA-seq) identified a *SEPT7-BRAF* fusion resulting from a pericentric inversion of chromosome 7. Subsequent testing of nine additional IFS-like pediatric sarcomas disclosed four additional tumors with *BRAF* rearrangements. Overall, these tumors occurred in patients 6 months to 16 years of age, and four out of the five occurred in the truncal region (one retroperitoneal, two pelvic, and one paraspinal), unlike typical IFS.

In 2017, Flucke *et al*. ^25^ described a spindle cell sarcoma presenting as a mitotically active, large pelvic soft tissue mass in a four-month-old and strongly expressing S100 by IHC. This lesion resembled IFS and, unlike malignant peripheral nerve sheath tumors, lacked SOX10 expression and retained H3K27me3. The tumor responded to adjuvant chemotherapy with ifosfamide, vincristine, and actinomycin D. FusionSeq revealed a *TFG-MET* fusion, validated by reverse transcription-polymerase chain reaction (RT-PCR) and FISH. After 12 months, the child had stable residual disease.

## Differential diagnosis

### Spindle cell rhabdomyosarcoma

**Figure 9.**
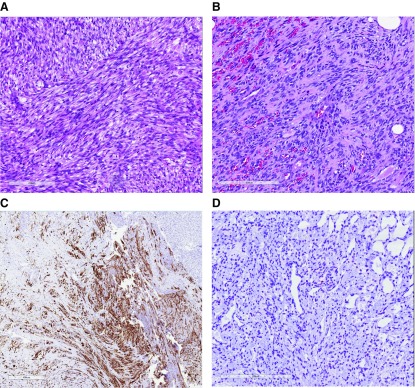
Spindle cell rhabdomyosarcoma. ( **A**) Some tumors like this spindle cell lesion contain spindle cells with easily recognizable rhabdomyoblasts with eosinophilic cytoplasm. ( **B**) Some tumors resemble fibrosarcoma and contain increased intercellular collagen and show less differentiation. ( **C**) Desmin staining is positive. ( **D**) Myogenin staining shows only rare positive nuclei, creating diagnostic confusion if not correlated with MyoD and desmin stains. ( **A**, **B**) Hematoxylin and eosin. ( **C**) Desmin immunostain. ( **D**) Myogenin immunostain. Scale bars attached. This figure uses an original image taken by DMP for publication.

Lest we forget, rhabdomyosarcoma is still by far the most common sarcoma in infants and children. For this single reason, it is important to always consider this diagnosis in spindle cell lesions, even those that do not have the typical features of embryonal rhabdomyosarcoma (ERMS). Interestingly, a recent study ^26^ suggests that some infantile rhabdomyosarcomas show a molecular profile differing from that of other rhabdomyosarcomas and resembling that of myofibroblastic tumors. These lesions, the so-called SCRMSs, are most frequently encountered in the paratesticular soft tissue, followed by the head and neck ^27^. They comprise fascicles of spindle cells with fusiform nuclei and indistinct eosinophilic cytoplasm, and variable fibrosis may be abundant (sclerosing rhabdomyosarcoma) ( Figure 9). SCRMS may resemble fibrosarcoma or leiomyosarcoma, making desmin and myogenin and myoD1 stains important parts of the workup of infantile fibrosarcomatous lesions. At close examination, however, cross-striations are more likely to be seen than with other rhabdomyosarcoma subtypes (personal observation). My own technique is to lower the condenser and increase the illumination before spending a little time looking under a high-power (40×) objective.

In children, SCRMS has been associated with improved outcome and has been considered a good prognostic subgroup ^28^. However, both of these factors are changing with new classification and genetic study. For one thing, SCRMS is now considered by WHO to represent a separate category of RMS, not a subgroup of ERMS. In our review of tumor from nine consecutive Children’s Oncology Group (COG) trials, Rudzinski *et al*. ^29^ found that, when analyzed by primary site, the outcome of SCRMS did not differ from that of typical ERMS, but there was one exception: parameningeal SCRMS had an outcome (23% event-free survival) inferior to that of ERMS.

It has been recognized that SCRMS is not a good diagnosis in adults ^27^, 2013, and one factor appears to be a distinctive *MYOD1* L122R mutation that characterizes these lesions ^30, 31^. This mutation seems to explain the aggressive behavior of these lesions and justifies separation of this histology as a distinct subtype. *MYOD1* mutation is not limited to adult SCRMS, as shown by Agaram *et al*. ^32^, who reported four out of five cases in children, all dying 12 to 36 months after diagnosis.

On the other hand, some infantile SCRMSs show recurrent *NCOA2* arrangements ^33^. In one study of infantile SCRMS ^33^, *SRF-NCOA2* and *TEAD1-NCOA2* studies were found, and another study ^34^ found recurrent *NCOA2* rearrangements as well as *VGLL2* rearrangements ( *VGLL2-CITED* in four tumors and *VGLL2-NCOA2* in two). Thus, *VGLL2-NCOA2* and alternate fusions appear to represent a distinct molecular event that leads to tumorigenesis in infants. None of the fusion-positive cases in this group developed metastatic disease, in contradistinction to the poor outcome of 10 out of 15 older patients with *MYOD1*-mutant positive SCRMS. This is the subgroup that appeared to have myofibroblastic properties in a study by Watson *et al*. ^26^.

## Dermatofibrosarcoma protuberans

Dermatofibrosarcoma protuberans (DFSP) is usually seen in adults and typically arises as plaque-like masses in the dermis and subdermis. It rarely occurs in children, although a variant lesion, giant cell fibroblastoma (GFC), is the more common presentation. Otherwise, DFSPs in children look similar to those in adults. Histologically, DFSP typically has a storiform pattern and strongly expresses CD34 but can transform into a fibrosarcoma pattern and lose CD34, thus creating overlap with the above entities.

*COL1A1-PDGFB* fusion is seen in both DFSP and GCF, but about 4% of lesions with typical histology and CD34 positivity are negative on routine screening ^35^. An investigation by Dadone-Montaudié *et al*. ^35^ of 21 FISH-negative cases revealed the classic *COL1A1-PDGFB* fusion in eight by RNA-seq. Eleven additional cases displayed novel *PDGFD* rearrangements involving fusions with either *COL6A3* or *EMILIN2*. That study confirms that alternate fusions may occur in DFSP and emphasizes the fact that negative *PDGFB* FISH can be misleading in otherwise typical cases.

## Calcifying aponeurotic fibroma

Calcifying aponeurotic fibroma (CAF) is a fibroblastic neoplasm with a predilection for the hands and feet of pediatric patients. These tumors typically show a biphasic morphology with bland fibroblasts merging with fibrocartilaginous nodules and osteoclasts, but limited biopsies may show only the fibroblastic pattern. S100 expression is seen in the cartilaginous foci.

A recent RNA-seq study by Puls *et al*. ^36^ found *FN1-EGF* fusions in a series of nine CAF cases. All tested were positive by either RT-PCR or FISH. EGF expression was seen by IHC in all of the CAF cases and none of the palmar/plantar fibromatoses or desmoids. Interestingly, EGF was also positive in six out of eight soft tissue chondromas, suggesting a possible relationship.

## Summary

The previous decade has provided much biological information regarding an important tumor group that preferentially affects infants and young children. These lesions have the potential to lead to deformation, limited mobility, and death, but as our knowledge has expanded, so have available treatment options. New information will undoubtedly lead to refinements in our knowledge of these lesions, their proper nosology, and the best treatment for affected patients.

Unanswered and still controversial points include the relationship of LPF-like neural tumors to NTRK-negative LPF and to IF. The most logical approach would be to lump all of these lesions under the rubric of “TRK-fusion associated tumor of intermediate malignant potential” to include both LPF-like neural tumors and IFs as well as NTRK1 fusion sarcomas. Regardless of nosology, all of these lesions, if unresectable, should be considered for treatment with a TRK inhibitor such as larotrectinib. A similar situation occurs with so-called NTRK fusion-positive “gastrointestinal stromal tumors”.

A similar situation applies to the relationship of PMMTI to CCSK and BCOR-related URCS. Like IF and cellular mesoblastic nephroma, CCSK and PMMTI are distinguished only by their renal or non-renal location, and URCS appears to be a high-grade version of PMMTI. With other renal sarcomas, such as primitive neurectodermal tumors, *CIC-DUX* fusion sarcomas, desmoplastic small round cell tumors, or synovial sarcomas, no attempt is made to separate renal from non-renal variants. If suitable targeted therapy becomes available for these neoplasms, it would seem to be prudent to treat them all similarly, regardless of location. As for separating CCSK and PMMTI, tumor grading has become standard practice, and so the logic for separation becomes tenuous if one applies common practice in other sarcomas such as leiomyosarcoma, synovial sarcoma, and liposarcoma.

The opposite situation occurs with SCRMS, in which tumors with similar morphology but widely diverse clinical behavior and biology are lumped into one group. To some degree, this situation results from the most recent WHO classification, which separated SCRMS from other RMS variants on the basis of its aggressive behavior and discrete morphology in adults. However, in children, it has been standard practice to consider SCRMS a prognostically favorable subtype of ERMS, and tumors with mixed SCRMS-ERMS histology occur frequently. Recent molecular findings have clarified this situation now that tumors with *MyoD* mutations versus *NCOA2/VGLL2* fusions have been described. It seems prudent now to consider SCRMS to be two prognostically and biologically separate subtypes of rhabdomyosarcoma, much as ERMS and *PAX-FOXO1* fusion-positive alveolar RMS have become.

There is still much to learn with infantile fibrous tumors, and findings in some tumors have yet to be elucidated. Undoubtedly, these will be the subject of future studies and trials of targeted agents.
